# Marine Chitinolytic *Pseudoalteromonas* Represents an Untapped Reservoir of Bioactive Potential

**DOI:** 10.1128/mSystems.00060-19

**Published:** 2019-06-18

**Authors:** Sara Skøtt Paulsen, Mikael Lenz Strube, Pernille Kjersgaard Bech, Lone Gram, Eva C. Sonnenschein

**Affiliations:** a Department of Biotechnology and Biomedicine, Technical University of Denmark, Lyngby, Denmark; University of British Columbia

**Keywords:** bioactivity, glycosyl hydrolases, *Pseudoalteromonas*, chitin

## Abstract

Chitin is the most abundant polymer in the marine environment and a nutrient-rich surface for adhering marine bacteria. We have previously shown that chitin can induce the production of antibiotic compounds in *Vibrionaceae*, suggesting that the discovery of novel bioactive molecules from bacteria can be facilitated by mimicking their natural habitat. The purpose of this study was to determine the glycosyl hydrolase (GH) profiles of strains of the genus *Pseudoalteromonas* to enable selection of presumed growth substrates and explore possible links to secondary metabolism. Genomic analyses were conducted on 62 pigmented and 95 nonpigmented strains. Analysis of the total GH profiles and multidimensional scaling suggested that the degradation of chitin is a significant trait of pigmented strains, whereas nonpigmented strains seem to be driven toward the degradation of alga-derived carbohydrates. The genomes of all pigmented strains and 40 nonpigmented strains encoded at least one conserved chitin degradation cluster, and chitinolytic activity was phenotypically confirmed. Additionally, the genomes of all pigmented and a few nonpigmented strains encoded chitinases of the rare GH family 19. Pigmented strains devote up to 15% of their genome to secondary metabolism, while for nonpigmented species it was 3% at most. Thus, pigmented *Pseudoalteromonas* strains have a bioactive potential similar to that of well-known antibiotic producers of the *Actinobacteria* phylum. Growth on chitin did not measurably enhance the antibacterial activity of the strains; however, we demonstrated a remarkable co-occurrence of chitin degradation and the potential for secondary metabolite production in pigmented *Pseudoalteromonas* strains. This indicates that chitin and its colonizers of the *Pseudoalteromonas* genus represent a so far underexplored niche for novel enzymes and bioactive compounds.

**IMPORTANCE** Infectious bacteria are developing and spreading resistance to conventional treatments at a rapid pace. To provide novel potent antimicrobials, we must develop new bioprospecting strategies. Here, we combined *in silico* and phenotypic approaches to explore the bioactive potential of the marine bacterial genus *Pseudoalteromonas*. We found that pigmented strains in particular represent an untapped resource of secondary metabolites and that they also harbor an elaborate chitinolytic machinery. Furthermore, our analysis showed that chitin is likely a preferred substrate for pigmented species, in contrast to nonpigmented species. Potentially, chitin could facilitate the production of new secondary metabolites in pigmented *Pseudoalteromonas* strains.

## Abstract (precis)

Infectious bacteria are developing and spreading resistance to conventional treatments at a rapid pace. To provide novel potent antimicrobials, we must develop new bioprospecting strategies. Here, we combined *in silico* and phenotypic approaches to explore the bioactive potential of the marine bacterial genus *Pseudoalteromonas*. We found that pigmented strains in particular represent an untapped resource of secondary metabolites and that they also harbor an elaborate chitinolytic machinery. Furthermore, our analysis showed that chitin is likely a preferred substrate for pigmented species, in contrast to nonpigmented species. Potentially, chitin could facilitate the production of new secondary metabolites in pigmented *Pseudoalteromonas* strains.

## INTRODUCTION

The increasing development and spread of antimicrobial resistance are a serious threat to human health, and humanity is in urgent need for novel antibiotics to treat infectious diseases. In particular, soil bacteria have been explored extensively as a source of antibiotics, and bacteria belonging to the phylum *Actinobacteria* have been a prolific source, providing two-thirds of all known microbial antibiotics (1). Exploring new environments is one strategy for finding novel compounds that are not (yet) affected by resistance. While promising bioactive molecules have been identified from marine organisms, particularly from the family *Pseudoalteromonadaceae* (2–5), the marine environment still remains an underexploited resource of novel bioactive compounds (6–8).

The exclusively marine genus *Pseudoalteromonas* constitutes, on average, 2 to 3% of the bacterial abundance in the upper ocean waters (9). *Pseudoalteromonas* strains are excellent biofilm formers and are often found in association with eukaryotic hosts, such as crustaceans or algae (10). As of 2018, 47 *Pseudoalteromonas* species had validly published names (11). The genus is phenotypically and phylogenetically divided into two main clusters that are differentiated by the ability to produce pigments and the lack thereof (12). The pigmented species produce an array of bioactive secondary metabolites, including violacein, indolmycin, and pentabromopseudilin, produced by Pseudoalteromonas luteoviolacea; prodigiosin, produced by P. rubra; bromoalterochromides, produced by P. piscicida; and tambjamines, produced by P. tunicata, P. flavipulchra, and P. maricaloris (13–16). In contrast, nonpigmented species have generally been explored as producers of unusual enzymatic activities (10).

On a global marine research expedition, we isolated strains of both pigmented and nonpigmented *Pseudoalteromonas* based on their antimicrobial activity (17); however, the nonpigmented strains did not retain antimicrobial activity following frozen storage (13). The genomes of four pigmented and three nonpigmented strains were sequenced and mined for biosynthetic gene clusters (BGCs) of secondary metabolites, revealing a large untapped potential in the pigmented strains (18). For most of the BGCs, the associated chemistry has not been elucidated, potentially because the BGCs are not expressed (e.g., are silent or cryptic) or are expressed at low levels under growth conditions hitherto used (13). Mimicking the natural growth substrate to induce bioactivity has been successful in the *Vibrionaceae*. Providing vibrios with chitin, the most abundant polymer in the marine environment, can enhance their antibacterial activity (19, 20) and can induce expression of their BGCs (21). Some *Pseudoalteromonas* species can also degrade chitin (18, 22), but little is known about their chitinolytic machinery and a possible influence on secondary metabolism. In the *Vibrionaceae*, specifically, Vibrio cholerae (23, 24), chitin degradation relies on the secretion of extracellular chitinases. In bacteria, the majority of chitinases belong to glycosyl hydrolase (GH) family 18 (25). Recently, chitinases belonging to GH family 19 have been discovered in a few groups of prokaryotes (26–30), and we have found that the genomes of 10 marine chitinolytic bacteria, including *Pseudoalteromonas*, all contain at least one GH19 chitinase (31).

The purpose of this study was to determine the chitinolytic abilities of species of the genus *Pseudoalteromonas* and explore possible links to their potential for secondary metabolite production. We used a genome sequence-guided approach combined with phenotypic assays to assess chitin degradation and antibacterial activity.

## RESULTS AND DISCUSSION

**FIG 1:**
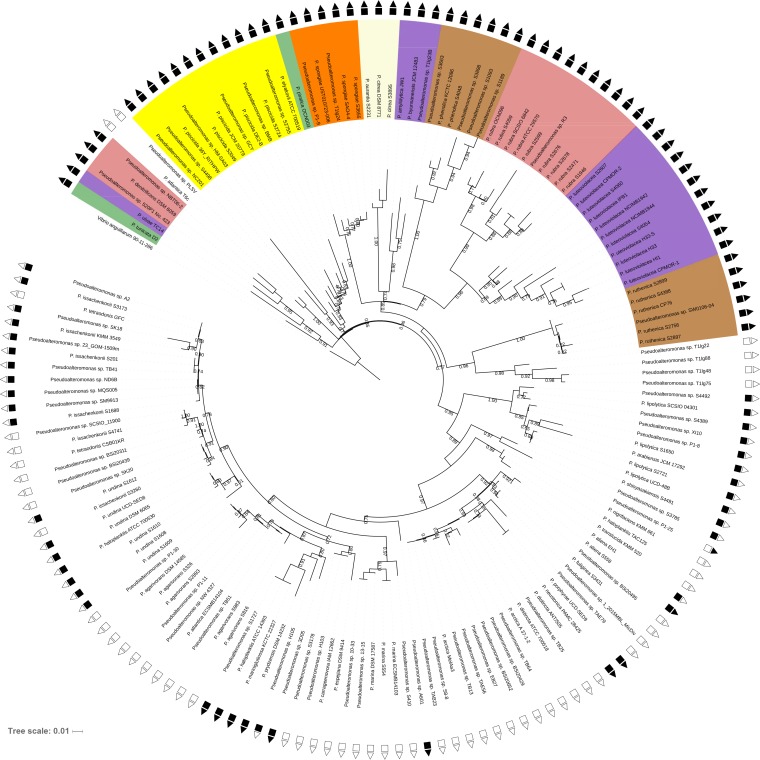
SNP-based phylogeny of 157 *Pseudoalteromonas* strains consisting of 50 of our isolates and 107 genomes downloaded from the NCBI database. Symbols are color coded, where black symbols represent that the feature is present in the genome and white symbols represent that the feature is absent (squares, chitinolytic genotype; triangles, GH19 chitinases are present in the genome). The pigmented strains are colored according to their pigmentation. The phylogenetic tree was constructed using the CSI Phylogeny web server, and V. anguillarum 90-11-286 (GenBank accession no. CP011460 and CP011461) was used as a root. Bootstrap values of >0.5 are included.

The average nucleotide identity (ANI) of 253 *Pseudoalteromonas* genomes obtained from isolates collected on a global marine expedition (17) and from the NCBI database was determined. The data set had a strong bias toward certain taxonomic subgroups being overrepresented among the total number of genomes. Therefore, in the case of an ANI of more than 98.3% (see Fig. S1 and S2 in the supplemental material), a representative for this phylogenetic subgroup was selected for further genomic analyses on the basis of sequence quality (Table S1). The resulting data set consisted of 157 strains covering 37 of the 47 species with standing in the prokaryote nomenclature (List of Prokaryotic Names with Standing in Nomenclature [LPSN] [11]). Single nucleotide polymorphism (SNP)-based phylogenetic analysis (Fig. 1) confirmed the previous clustering based on 16S rRNA gene analysis and separated the *Pseudoalteromonas* strains into two phylogenetic groups that correlated with the pigmented and nonpigmented phenotypes (13), with the exception of two nonpigmented strains (P. atlantica T6c and *Pseudoalteromonas* sp. strain PLSV), which clustered in the pigmented group. Sixty-two strains belonged to pigmented species, and 95 strains belonged to nonpigmented species. Pigmented species had a genome size of 5.2 ± 0.6 Mb, while nonpigmented species had smaller genomes of 4.4 ± 0.4 Mb (Table S1). In general, the phylogenetic diversity was higher in the pigmented species, whereas nonpigmented species were phylogenetically more similar (Fig. 1; Fig. S2). This could indicate a higher selection pressure for pigmented strains or a more recent evolution of nonpigmented strains. Based on the ANI analysis (Fig. S2), several of the strains, particularly those within the pigmented group, could belong to novel species. This is in agreement with the findings of a recent study by Busch et al. (5), in which the authors noted that considerable species-level diversity has yet to be described in the *Pseudoalteromonas* genus. Furthermore, the ANI analysis also enabled some unclassified strains to become assigned to species. The whole genomes of 10 taxonomically classified species have not yet been sequenced, and therefore, these species were not included in the analysis and some unassigned strains could belong to these species. The ANI analysis also demonstrated that the ANI of some strains were <95% and, thus, that these strains should be reclassified as other species (32).

10.1128/mSystems.00060-19.1 FIG S1 Correlation of percent ANI to the percentage of isolates used for inferring the cutoff percent ANI value of identical isolates. Download FIG S1, PDF file, 0.1 MB. Copyright © 2019 Paulsen et al. 2019 Paulsen et al. This content is distributed under the terms of the Creative Commons Attribution 4.0 International license.

10.1128/mSystems.00060-19.2 FIG S2 Heat map of average nucleotide identity (ANI) analysis of 157 *Pseudoalteromonas* strains. The color-coded bar on the left indicates the pigmentation status. Download FIG S2, PDF file, 0.4 MB. Copyright © 2019 Paulsen et al. 2019 Paulsen et al. This content is distributed under the terms of the Creative Commons Attribution 4.0 International license.

10.1128/mSystems.00060-19.3 TABLE S1 List of 50 *Pseudoalteromonas* strains isolated during the Galathea 3 expedition and 107 *Pseudoalteromonas* genomes obtained from NCBI, the species, their pigmentation, the genome size (in megabases), the percentage of the genome allocated to BGCs, and the NCBI accession number. Download Table S1, PDF file, 0.4 MB. Copyright © 2019 Paulsen et al. 2019 Paulsen et al. This content is distributed under the terms of the Creative Commons Attribution 4.0 International license.

The high level of dissimilarity between some of the species could even suggest that the *Pseudoalteromonas* genus potentially represents multiple genera, as was the case for the genus *Algicola*, consisting of the two species Algicola bacteriolytica and A. sagamiensis, which belonged to the *Pseudoalteromonas* genus until 2007 (33). Since ANI serves as a genomic measurement only for species delineation and not for genus delineation, this is only speculative. Nonetheless, the ANI analysis clearly visualized a problem with species naming within the *Pseudoalteromonas* genus, as some strains were not correctly assigned to a species. For example, P. undina DSM 6065 and P. haloplanktis ATCC 700530 share 98% ANI similarity, thus exceeding the species cutoff for ANI of ≥ 95% (32). Also, P. mariniglutinosa KCTC 22327 and P. haloplanktis ATCC 700530 have 99% ANI similarity and P. neustonica PAMC 28425 and P. porphyrae UCD-SED9 share 99% ANI similarity, indicating that these are the same species. Similar issues with species delineation have been reported in the *Pseudomonas* genus (34), as well as on a much larger scale for prokaryotes (35).

### Genes encoding chitin degradation machinery are common in pigmented *Pseudoalteromonas* strains.

Of the 157 strains, 102 strains had the genomic capacity to degrade chitin (Fig. 1). The chitin-degrading genotype matched the chitin-degrading phenotype (Table S2). All 62 genomes of pigmented strains encoded chitinase genes from both the GH18 and GH19 families. Forty-five percent (40 of 95) of the genomes of the nonpigmented strains encoded chitinase genes of the GH18 family. Of those, only 10 genomes also encoded chitinases from the GH19 family.

10.1128/mSystems.00060-19.4 TABLE S2 Chitin degradation on agar plates containing colloidal chitin and inhibition of Vibrio anguillarum by *Pseudoalteromonas* strains on media supplemented with four different carbon sources: glucose, *N*-acetylglucosamine, colloidal chitin, or crystalline chitin. +, inhibition; −, no inhibition. Download Table S2, PDF file, 0.3 MB. Copyright © 2019 Paulsen et al. 2019 Paulsen et al. This content is distributed under the terms of the Creative Commons Attribution 4.0 International license.

**FIG 2:**
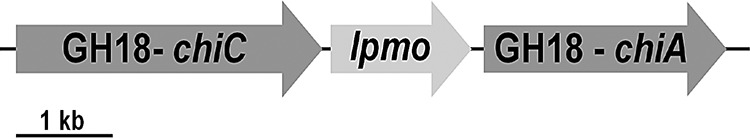
The chitin degradation cluster (CDC) present in all chitinolytic *Pseudoalteromonas* strains. The CDC consists of a GH18 chitinase of the *chiC* type, a lytic polysaccharide monooxygenase (*lpmo*), and a GH18 chitinase of the *chiA* type.

The genomes of pigmented strains encoded, on average, 5.2 GH18 chitinases, while the nonpigmented chitinolytic strains encoded, on average, 2.1 GH18 chitinases. Hence, the pigmented strains encoded significantly more GH18 chitinases than nonpigmented chitinolytic strains, with an average of 2.4 times more GH18 chitinases (*P* < 0.0001) being found in pigmented strains. A three-gene chitin degradation cluster (CDC) (Fig. 2), encoding a GH18 chitinase of the ChiC type, a lytic polysaccharide monooxygenase (LPMO), and a GH18 chitinase of the ChiA type, was found in all chitinolytic strains and is likely a conserved feature in chitin-degrading *Pseudoalteromonas* strains independent of pigmentation. The CDC was initially described in 2002 by Tsujibo et al. at a time when LPMOs had not been described (36). Today, it is known that chitin-associated LPMOs are abundant in prokaryotes (37) and facilitate the breakdown of chitin by catalysis of the oxidative cleavage of glycosidic bonds (38–40). The genomes of the majority of pigmented strains belonging to the species P. luteoviolacea, P. rubra, P. phenolica, and P. citrea encoded two CDCs.

The conservation of GH19 chitinases in all pigmented strains indicates an important role of this enzyme in this group. GH19 chitinases are mostly known from plants and are rare and thus understudied in bacteria and fungi (37, 41–43). In comparison, a recent analysis of 40 *Micromonospora* strains whose whole genomes have been sequenced found that all genomes carried the *chiC* gene, encoding the GH18 chitinase, but none contained the GH19-encoding genes (44). In plants, chitinases mainly serve as antifungal agents, and some studies have suggested that this could be one of the functions of bacterial GH19 chitinases (27, 30, 41, 45). Antifungal activity is, however, not limited to GH19 chitinases, as some GH18 chitinases also display antifungal activities (46, 47). The first GH19 chitinase described in *Pseudoalteromonas* was antifungal, but it also hydrolyzed colloidal and crystalline chitin (26). Thus, the functional differences between the GH18 and GH19 chitinases remain unresolved.

### Pigmented and nonpigmented *Pseudoalteromonas* strains are distinct in their GH profiles.

**FIG 3:**
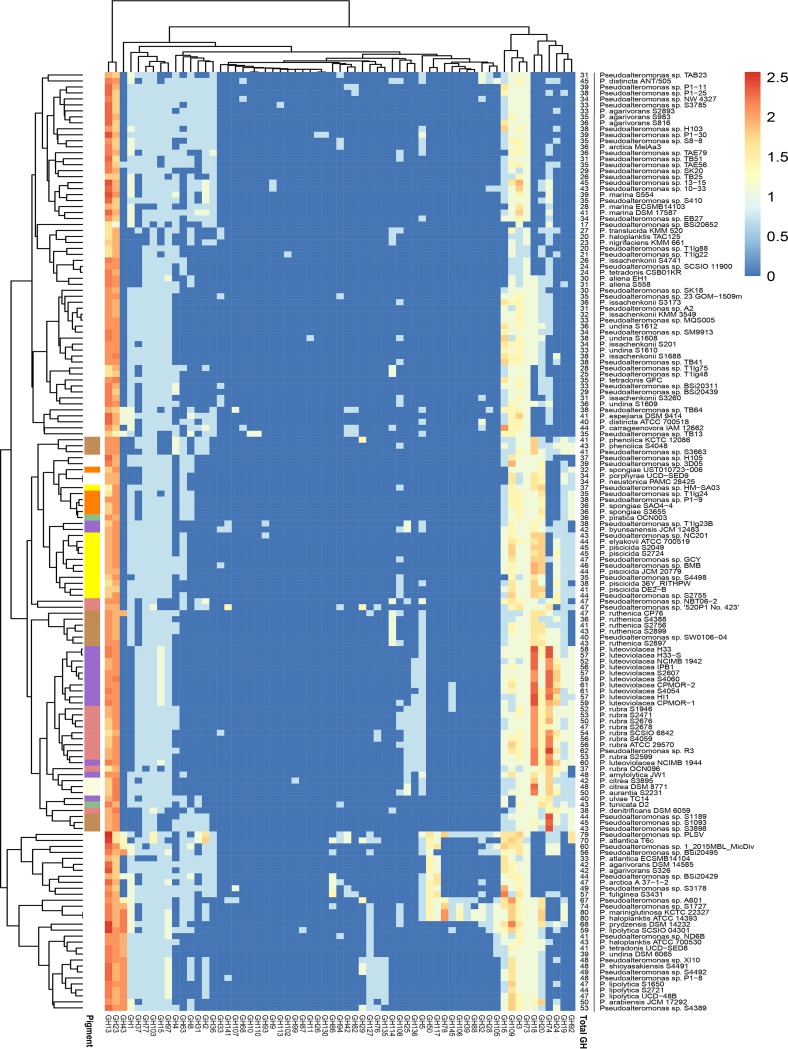
Functionally clustered heat map based on the predicted glycosyl hydrolases (GH) of the 157 *Pseudoalteromonas* strains. The key displays the log-transformed gene counts of GHs.

A clustered heat map was generated based on the predicted GH profiles of the strains (Fig. 3). Pigmented and nonpigmented strains contained, on average, 47 ± 8 and 40 ± 13 GHs, respectively. The functional clustering divided the strains into two groups, with one consisting solely of nonpigmented strains and the other consisting of both pigmented and nonpigmented strains, and subclustering correlated with the phylogeny and pigmentation. In total, 63 out of 156 entries of GHs described in the CAZy database by the start of 2019 (http://www.cazy.org/) were represented in the strains. Of the 63 different GH families identified, 60 GH families were represented in nonpigmented strains, whereas only 38 GH families were found in pigmented strains. The Shannon index was higher in the pigmented strains (*P* < 0.001), but the variation was 8-fold larger in the nonpigmented strains. This suggests that even though the average pigmented strain genome based on gene counts contains more GHs than the average nonpigmented strain genome, the GH profiles of the nonpigmented strains are more heterogeneous than those of the pigmented strains.

Interestingly, the functional clustering detected a pattern of co-occurrence between the GH19 chitinase and the GH92 mannosidase, meaning that of the 71 genomes containing at least one GH19 gene, 71.8% also contained a GH92 gene (*P* < 0.00001). In the mycoparasitic fungus Trichoderma harzianum, chitinases and a GH92 mannosidase were significantly upregulated in response to pathogenic fungi (48). Since chitin and mannose are components of the fungal cell wall, this could indicate that the natural role of bacterial GH19s is indeed antifungal, possibly in combination with GH92.

**FIG 4:**
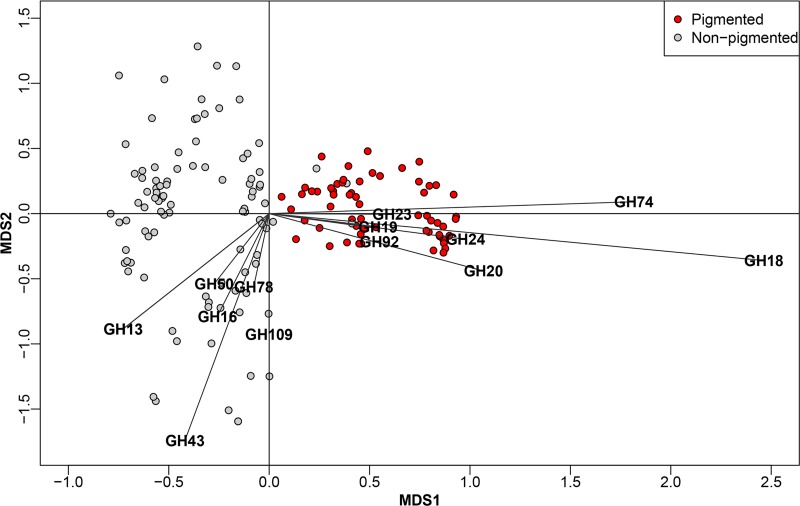
Multidimensional scaling (MDS) plot of pigmented (red dots) and nonpigmented (gray dots) *Pseudoalteromonas* strains based on their glycosyl hydrolase (GH) profile. GHs with loadings of more than ±0.5 are shown in the graph.

To evaluate if the GH profiles differed on the basis of pigmentation status, multidimensional scaling (MDS) was conducted. MDS clearly separated pigmented and nonpigmented strains into two groups on the basis of their GH profiles (Fig. 4). This is in accordance with analysis of similarities (ANOSIM) showing a significant effect of pigmentation in multivariate space (*P* < 0.001) and suggesting that pigmentation status explains ∼50% of the GH profile (*r*^2^ = 0.50). Listed in order of the most significant contribution, GH18, GH74, GH20, GH24, GH19, GH92, and GH23 had the major impact of this divergence for pigmented strains (loadings < 0.5). For nonpigmented strains, GH13, GH43, GH50, GH16, GH78, and GH109 had a major divergent impact on the GH profile (loadings > −0.5) (Table S3). Thus, the GH18 chitinase was the strongest contributor to the GH profiles of pigmented strains, as it was present in all genomes and was present at a higher abundance than it was in nonpigmented strains. Also, the chitinolytic GH19s and GH20s contributed prominently to the separation of the GH profiles of pigmented strains, proposing that chitin degradation is a key physiological trait in pigmented *Pseudoalteromonas* strains. The loading coordinates of GH19 and GH92 were very similar, confirming their co-occurrence, as discussed above. For nonpigmented strains, GH13 was the strongest contributor to the diversification from pigmented species. The contribution of GH13 in nonpigmented strains is reflected by a higher gene count compared to that in pigmented species. GH43, GH50, GH16, GH78, and GH109 also contributed to the intergroup diversity of the nonpigmented GH profiles, and all are enzymes that are active on marine algal polysaccharides, such as agar, porphyrin, carrageenan, starch, and glucan. The building blocks of algal polysaccharides are relatively few, but they have a high structural complexity, as they can be linked in almost infinite ways and are also often subject to acetylation, methylation, or sulfation (49). The high diversity of GH families in nonpigmented species could indicate that this group favors the utilization of algal polysaccharides. Pigmented strains also have the genetic capacity to utilize algal components, and many nonpigmented strains can degrade chitin; however, the conserved chitinolytic machinery in pigmented strains indicates a specialized adaptation to chitin utilization.

10.1128/mSystems.00060-19.5 TABLE S3 Loadings associated with multidimensional scaling plot in Fig. 4. Loadings plotted in Fig. 4 are represented in bold. Download Table S3, PDF file, 0.2 MB. Copyright © 2019 Paulsen et al. 2019 Paulsen et al. This content is distributed under the terms of the Creative Commons Attribution 4.0 International license.

The general perception of the genus *Pseudoalteromonas* is that pigmented species produce many bioactive secondary metabolites, whereas nonpigmented species produce a larger amount of hydrolytic enzymes (10). The results presented herein partially support this assumption, as the diversity of GHs was higher in nonpigmented strains, but not on a gene count basis. While having less diverse GH profiles, the genomes of pigmented strains contained, on average, at least the same number of GHs as nonpigmented strains or higher number of GHs than nonpigmented strains.

### *Pseudoalteromonas* strains devote up to 15% of their genome to the biosynthesis of secondary metabolites.

**FIG 5:**
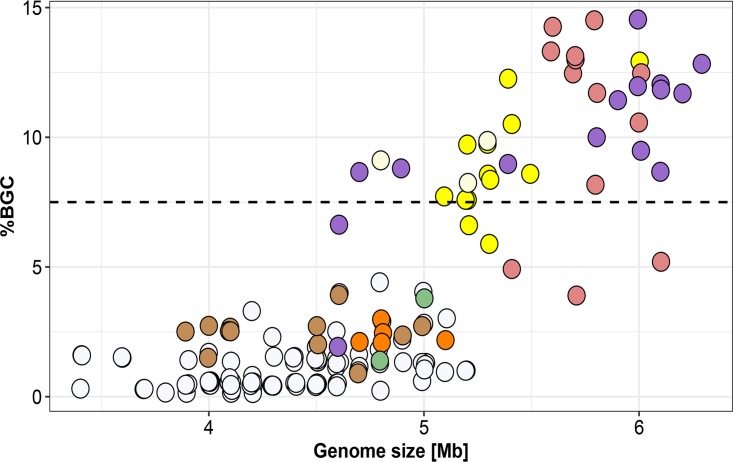
Proportion of biosynthetic gene clusters (BGCs) in the *Pseudoalteromonas* genomes according to genome size. Each circle represents one genome and is color coded based on pigmentation. The data have been jittered to account for overlying points.

The genomic bioactive potential of all *Pseudoalteromonas* strains was compared to that of well-known secondary metabolite producers (50) by calculating the proportion of the genome dedicated to the biosynthesis of secondary metabolites (percent BGC) (Fig. 5; Table S1). Mining the genomes using the antiSMASH tool, we found that the genomes of pigmented strains dedicated, on average, 7.6% ± 4.2% to BGCs and that those of nonpigmented strains dedicated, on average, 1.1% ± 0.9%. In comparison, the average prokaryote devotes 3.7% ± 3.1% of its genome to BGCs (50). There was a strong linear correlation between genome size and percent BGC, which was especially pronounced in the pigmented strains (*r*^2^ = 0.60, *P* < 0.00001, slope = 5.18) compared to the nonpigmented strains (*r*^2^ = 0.13, *P* < 0.001, slope = 0.81), meaning that for every gigabase increase in genome size, a further 5.2% of the genome is dedicated to BGCs for pigmented species. The genomes of pigmented species encoded, on average, 6.8 times more BGCs (*P* < 0.00001) than those of nonpigmented species did. Thirty-six strains devoted more than 7.5% (50) of their genomes to BGCs, with the highest percentage of BGCs being found in P. luteoviolacea and P. rubra strains (12% to 15%). Thus, their genetic potential for the production of secondary metabolites is equal to that of species generally well-known for the production of secondary metabolites, including those in the genera *Streptomyces*, *Myxococcus*, *Sorangium*, and *Burkholderia* (50). It is also comparable to that in species from the marine actinomycete genus *Salinispora*, devoting approximately 10% of the investigated genomes to secondary metabolism (51, 52).

Genomic analysis of representatives of each pigmented species and three unclassified pigmented strains using antiSMASH found that most of the predicted BGCs showed little to no homology to known and characterized bioactive compounds (Table 1). Many of these BGCs may represent a novel chemistry or may encode novel homologs of the compound classes (13). Bacteria from the phylum *Actinobacteria* have been the cornerstone of the antibiotic era, as two-thirds of all known microbial antibiotics originate from these Gram-positive bacteria (1, 53). They remain a focal point of antibiotic research, since genome mining has revealed the presence of many potentially silent or cryptic BGCs in their genomes (54). Likewise, our study demonstrates that pigmented *Pseudoaltermonas* species could be a potent and promising source of new antimicrobials.

**TABLE 1:** Total number of predicted BGCs in a representative selection of pigmented species, including the most similar known BGCs according to percent gene similarity^*a*^

Species and strain	Total no. of predicted BGCs	Most similar known BGC (% gene similarity)
P. luteoviolacea S2607	23	Violacein (80%), kalimantacin (10%), vibriobactin (18%), griseobactin (17%), cystobactamide (8%)
P. spongiae S3655	5	Arylpolyene (35%), flexirubin (5%), desferrioxamine B (60%)
P. phenolica S1189	6	Xenocyloins (25%), vulnibactin (25%)
P. rubra S4059	25	Indigoidine (40%), turnerbactin (15%), kalimantacin/batumin (10%)
*Pseudoalteromonas* sp. strain S4498	9	Serobactins (15%)
P. piscicida S2049	18	Griseobactin (23%), feglymycin (10%), bromoalterochromides (14%), alterochromides (90%)
P. ruthenica S2897	4	Arylpolyene (40%), desferrioxamine B (60%)
P. aurantia S2231	17	Kalimantacin/batumin (10%), pyoverdine (1%)
P. citrea S3895	17	Pyoverdine (1%), turnerbactin (15%), pyoverdine (1%), zeamine (17%)
P. ulvae TC14	4	APE_Vf (40%), violacein (80%)
P. piratica OCN0013	3	APE_Vf (40%), desferrioxamine B (60%)
*Pseudoalteromonas* sp. strain 520P1	7	Violacein (80%), pyoverdine (2%), taxlllaid (6%), eicosapentaenoic acid-like compound (18%)
*Pseudoalteromonas* sp. strain HM SA03	11	Bromoalterochromides (14%), hectochlorin (37%), alterochromides (100%), turnerbactin (15%)
P. amylolytica JW1	16	Violacein (80%), staphylobactin (12%)
P. denitrificans DSM 6059	10	APE_Vf (45%), eicosapentaenoic acid-like (18%), bacillibactin (23%)
P. tunicata D2	5	Violacein (80%), prodigiosin (12%), desferrioxamine B (50%)
P. byunsanensis JCM 12483	14	Taxlllaid (4%), staphylobactin (12%), bromoalterochromides (14%), violacein (80%)
P. elyakovii ATCC 700519	18	Bacillibactin (60%), alterochromides (95%)

a BGCs were predicted and percent gene similarity was defined by use of the antiSMASH tool.

### Enhanced antibacterial activity could not be detected on chitin.

In some *Vibrionaceae*, BGCs are upregulated when the bacteria are grown on chitin, and the finding of an elaborate chitinolytic machinery in *Pseudoalteromonas* led us to determine the antibacterial activity of the strains when grown on four different carbon sources (glucose, *N*-acetylglucosamine [NAG], colloidal chitin, and crystalline chitin) mimicking a natural chitinous habitat (55). Of the 50 strains, all 24 pigmented strains and one nonpigmented strain (strain S558) were inhibitory toward the target pathogen, in agreement with previous results (13); however, inhibition was independent of the carbon source (Table S2). Thus, the overall antibacterial activity was not enhanced on chitinous media compared to glucose media, as has been seen for strains of the *Vibrionaceae* family (20). The assay was not sensitive enough to quantify the antibacterial activity, and chitin could potentially still enhance the response of a metabolite cluster that is also expressed without addition of chitin. This was seen for the antibiotic andrimid in Vibrio coralliilyticus S2052 when grown on chitin compared to glucose (19). Also, growth on chitin may alter the expression of BGCs encoding bioactivities other than antibacterial activity.

### Conclusion.

Here, we demonstrate that chitin is likely an important carbon source for pigmented *Pseudoalteromonas* species, as chitin hydrolysis is a key trait in this group of bacteria. Pigmented *Pseudoalteromonas* strains and a few nonpigmented strains contain GH19 chitinases, which are not common in bacteria. In plants, the main role of GH19 chitinases is antifungal, and the finding of a co-occurring mannosidase of the GH92 family suggests that the fungal cell wall could be the target of GH19 chitinases in bacteria as well. Nonpigmented *Pseudoalteromonas* strains have an extended algal polysaccharide-degrading GH profile compared to pigmented strains; in contrast, their genomic potential for producing bioactive compounds was much lower. The fact that pigmented species devote up to 15% of their genome to BGCs qualifies this genus to be in the same antibiotic-producing league as the well-known *Actinobacteria*, with the benefit of being less explored. We hypothesized that growth on chitin would enhance the bioactivity of the strains, but, when measured as antibiotic activity, this was not confirmed. However, this study serves as an extensive basis for ecology-based bioprospecting of secondary metabolites and hydrolytic enzymes and has uncovered a promising genomic potential for producing novel antimicrobial compounds in pigmented *Pseudoalteromonas*.

## MATERIALS AND METHODS

### *Pseudoalteromonas* genomes.

One hundred sixty-five *Pseudoalteromonas* genomes available in the NCBI database in May 2018 and 88 *Pseudoalteromonas* genomes (see below) from our global Galathea collection (17) were included. To avoid bias in the data set due to an uneven distribution of the number of strains per species and to exclude very similar isolates, an average nucleotide identity (ANI) analysis using the Python module pyani (56) was conducted using all 253 genomes to eliminate those sharing an ANI of greater than 98.3%. This cutoff was inferred by visually inspecting a plot of the number of isolates versus the ANI value, in which there was a notable elbow at 98.3% identity (see Fig. S1 in the supplemental material). Subsequently, 107 genomes from NCBI and 50 genomes sequenced in the present study were included in the bioinformatic analyses (Table S1). The data set included 15 pigmented species (62 strains) and 23 nonpigmented species (95 strains). These numbers are approximate, as some strains were not assigned to any species but clustered within either the pigmented or the nonpigmented group.

### Isolation of genomic DNA and genome sequencing.

Ninety-eight *Pseudoalteromonas* strains were isolated during the Galathea 3 global marine research expedition (17). They were isolated from seawater or swab samples from biotic or abiotic surfaces and selected on the basis of their antibacterial activity against the fish pathogen Vibrio anguillarum. The origins and coordinates of isolation can be found in the work of Gram et al. (2010) (17). The genomes of 12 strains were sequenced as part of a previous study (18, 57), and we were unable to purify DNA from 10 strains. Thus, 76 new genomes were sequenced as part of the present study. High-purity genomic DNA was extracted using a NucleoSpin tissue kit (catalog number 740952; Macherey-Nagel) including an RNase treatment step. For half of the strains, we had difficulties extracting high-purity DNA; therefore, a Qiagen Genomic-tip 20/G kit (catalog number 10223; Qiagen) and a genomic DNA buffer set (catalog number 19060; Qiagen) were used for extraction instead. Quantification was done using a DeNovix DS-11+ spectrometer (DeNovix, USA) and Qubit (v2.0) analyzer (Invitrogen, United Kingdom). Genomes were sequenced at the Novo Nordisk Center for Biosustainability (Technical University of Denmark, Lyngby, Denmark) using 150-bp paired-end sequencing on an Illumina NextSeq platform. Genomes were assembled using CLC Genomics Workbench software (v8; CLC bio, Aarhus, Denmark), and contig-based draft genomes were obtained; all had genome coverage of 105-fold or higher.

### Phylogenetic analysis.

A phylogenetic tree of the 157 genomes was generated with the SNP-based procedure CSI Phylogeny (http://cge.cbs.dtu.dk/services/CSIPhylogeny/) (58). Pseudoalteromonas luteoviolacea DSM 6061^T^ (WGS accession no. GCF_001625655.1) was used as a reference genome, and Vibrio anguillarum 90-11-286 (GenBank accession no. CP011460 and CP011461) was used as the outgroup. The maximum likelihood phylogenetic tree was visualized using iTOL (https://itol.embl.de/) (59).

### Bioinformatic analyses.

The genomes sequenced in the present study were annotated using the Rapid Annotation using Subsystem Technology (RAST) (60). The presence of a conserved chitin degradation cluster (CDC) was investigated using MultiGeneBlast (61) with the CDC query from (36). The lytic polysaccharide monooxygenase (LPMO) of the CDC was identified using Pfam (https://pfam.xfam.org/).

The total numbers of GHs encoded in each genome was predicted using Hidden Markov Model (HMM) searches against local versions of the dbCAN (62). A heat map of the GH profiles was constructed in R (v3.4.2) using the package pheatmap (63), in which the profiles were clustered by complete linkage on the log-transformed values for ease of visualization. The average GH18 gene content in pigmented versus nonpigmented chitinolytic strains was tested with a *t* test. A co-occurrence pattern between GH19 and GH92 was tested with a Fisher exact test. To visualize the differences in GH profiles based on pigmentation, a metric multidimensional scaling (MDS) analysis on a count matrix with the total number of predicted protein sequences per GH family was conducted in R using Bray-Curtis distances. Loadings with values of more than ±0.5 were plotted in the MDS plot. Analysis of similarities (ANOSIM) was used to test whether the pigmentation status was predictive of the GH gene profile in a multivariate context. To test if pigmentation status was associated with GH profile diversity, the Shannon index was calculated and tested with a *t* test.

All 157 genomes were analyzed for secondary metabolite gene clusters using the antiSMASH (v4.0) tool (64). The sizes of the predicted BGCs relative to the genome size were calculated for each strain (in nucleobases) and compared to the total size of the genome using linear regression. The percentage of the genome dedicated to BGCs in pigmented versus nonpigmented strains was tested with a *t* test.

### Chitin degradation.

To determine if the chitin-degrading genotype resulted in a phenotype, the 50 strains from the Galathea collection were tested for chitin degradation. Strains were grown with aeration at 25°C and 200 rpm overnight in marine broth (MB 2216; Difco BD). Two microliters of each culture was inoculated in duplicate on chitin agar, consisting of 1.5% agar, 2% sea salt (catalog number S9883; Sigma), 0.3% Casamino Acids, and 0.2% colloidal chitin (catalog number C7170; Sigma). Colloidal chitin was prepared as described previously (20). Plates were inspected daily for 10 days, and chitin degradation was determined by the appearance of a clearing zone around the colonies.

### Screening for antibacterial activity.

The antibacterial activity of the 50 Galathea strains against the fish pathogen V. anguillarum 90-11-286 (65) was tested using the agar-based assay described previously (20) with either 0.2% glucose, 0.2% *N*-acetylglucosamine (NAG), 0.2% colloidal chitin, or 0.2% crystalline chitin as the carbon source, in addition to 0.3% Casamino Acids. Bacterial strains were grown at 25°C and 200 rpm overnight in MB. Two microliters of each culture was inoculated in duplicate on the four media. On each plate, bacteria were inoculated in rows of four strains with a horizontal and a vertical distance of 25 mm from each other. Strains were allowed 2 days of growth before 2 μl of an overnight culture of the target strain, V. anguillarum 90-11-286, grown in MB was inoculated at a distance of 5 mm from the *Pseudoalteromonas* strains. The plates were incubated at 25°C, and the colony growth of the target strain was assessed 48 h after the target strain had been inoculated.

### Data availability.

The 76 new draft genomes are available at the National Center for Biotechnology Information (NCBI) database under accession numbers PNBS01 to PNEL01. All accession numbers are listed in Table S1. The strains are available upon request.
