# Supplementary figures and images for: Marine Chitinolytic Pseudoalteromonas Represents an Untapped Reservoir of Bioactive Potential
Source: mSystems. 2019 Jun 18;4(4):e00060-19. doi: 10.1128/mSystems.00060-19 (PMC6581688; doi:10.1128/mSystems.00060-19)

Fig S1

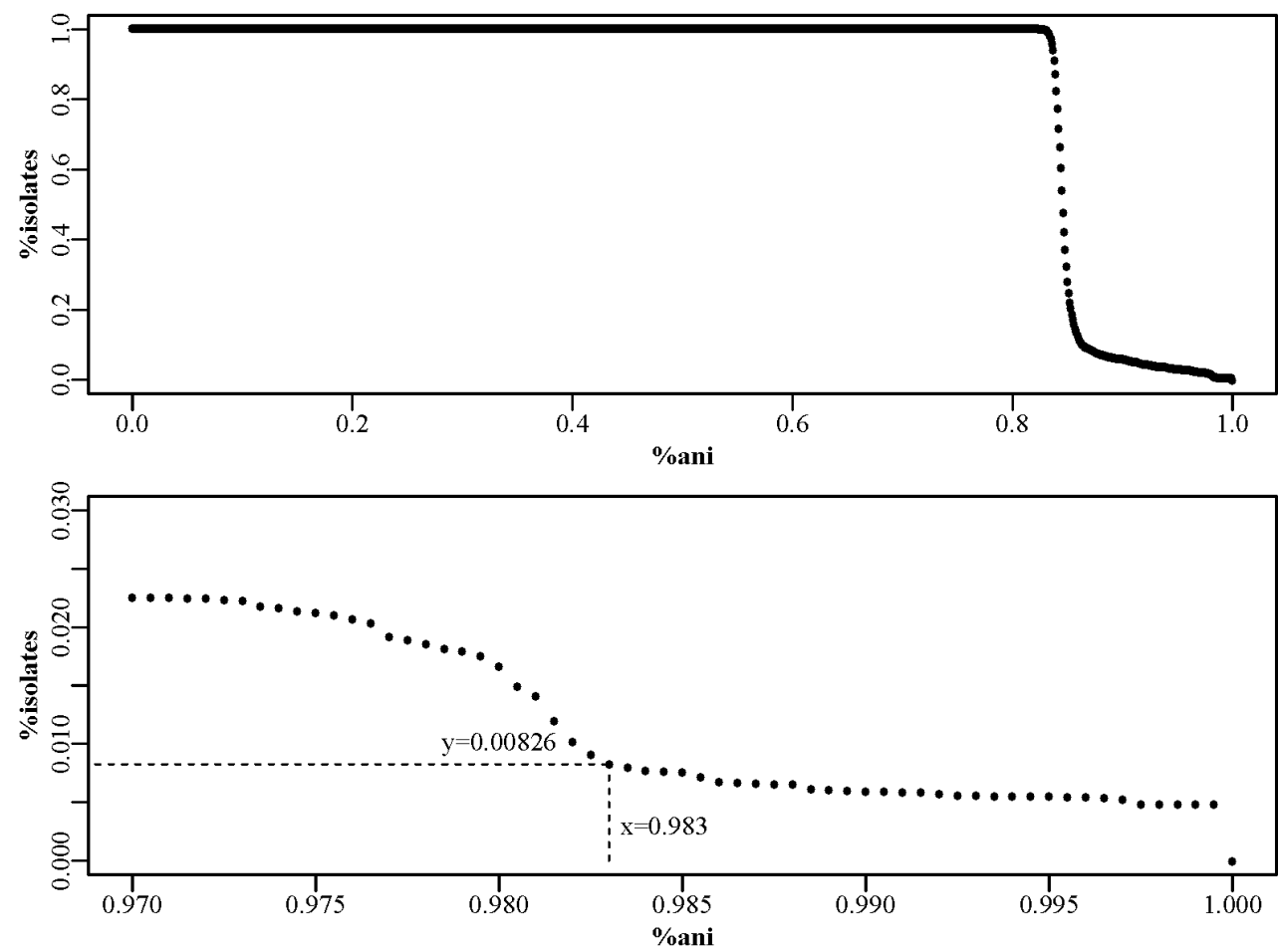

Supplement: FIG S1 [file mSystems.00060-19-sf001.pdf]
