# Supplementary material for: Marine Chitinolytic Pseudoalteromonas Represents an Untapped Reservoir of Bioactive Potential
Source: mSystems. 2019 Jun 18;4(4):e00060-19. doi: 10.1128/mSystems.00060-19 (PMC6581688; doi:10.1128/mSystems.00060-19)
Supplement: TABLE S1 [file mSystems.00060-19-st001.pdf]

Table S1

Species and strain name	Pigment	Genome size (Mb)	% BGC of genome	Accession/RefSeq
P. tunicata D2	+	5.0	3.79	GCF_002310815.1
P. ulvae TC14	+	4.6	1.92	GCF_002156545.1
Pseudoalteromonas sp. '520P1 No. 423'	+	5.4	4.92	GCF_001269985.1
P. denitrificans DSM 6059	+	6.1	5.2	GCF_900112265.1
Pseudoalteromonas sp. NBT06-2	+	5.7	3.9	GCF_002276045.1
Pseudoalteromonas sp. BMB	+	5.3	9.75	GCF_001709235.1
P. piscicida DE2-B	+	5.3	8.56	GCF_002208135.1
Pseudoalteromonas sp. HM-SA03	+	5.2	9.72	GCF_002289345.1
Pseudoalteromonas sp. GCY	+	5.4	10.51	GCF_002591815.1
P. piscicida 36Y_RITHPW	+	5.1	7.72	GCF_002744175.1
P. piscicida S2049	+	5.3	8.36	PNEF00000000
P. piscicida S2724	+	5.2	6.61	GCF_000967575.1
P. elyakovii ATCC 700519	+	5.5	12.92	GCF_000814665.1
Pseudoalteromonas sp. NC201	+	5.4	12.26	GCF_002850255.1
P. piscicida JCM 20779	+	5.5	8.59	GCF_000238315.3
Pseudoalteromonas sp. S2755	+	5.2	7.59	PNDS00000000
Pseudoalteromonas sp. S4498	+	5.3	5.89	PNCO00000000
P. piratica OCN003	+	4.8	1.39	GCF_000788395.1
Pseudoalteromonas sp. P1-9	+	4.8	2.98	GCF_001399975.1
Pseudoalteromonas sp. T1lg24	+	4.7	2.11	GCF_002964745.1
P. spongiae S3655	+	4.8	2.45	PNBZ00000000
P. spongiae SAO4-4	+	5.1	2.18	GCF_002814155.1
P. spongiae UST010723-006	+	4.8	2.08	GCF_000238255.3
P. citrea DSM 8771	+	5.3	9.86	GCF_000238375.2
P. aurantia S2231	+	5.2	8.24	PNCL00000000
P. citrea S3895	+	4.8	9.11	PNBW00000000
P. byunsanensis JCM 12483	+	4.7	8.66	GCF_001854475.1
P. amylolytica JW1	+	4.9	8.8	GCF_001854605.1
Pseudoalteromonas sp. T1lg23B	+	4.6	6.63	GCF_002964665.1
Pseudoalteromonas sp. S3663	+	4.7	0.9	SAMN08296079
Pseudoalteromonas sp. S3898	+	4.6	3.92	SAMN08296066
P. phenolica S4048	+	5.0	2.72	PNBV00000000
Pseudoalteromonas sp. S1093	+	4.5	2.02	PNEC00000000
Pseudoalteromonas sp. S1189	+	4.5	2.72	PNCM00000000
P. phenolica KCTC 12086	+	4.9	2.36	GCF_001444405.1
Pseudoalteromonas sp. R3	+	5.8	11.71	GCF_001282135.1
P. rubra OCN096	+	5.8	8.17	GCF_001188445.1
P. rubra S1946	+	5.6	14.26	SAMN08389157
P. *rubra* S2471	+	5.7	12.46	GCF_000967655.1
P. rubra S2599	+	5.6	13.31	PNCJ00000000
P. rubra S2676	+	5.7	13	PNCI00000000

P. rubra S4059	+	5.8	14.51	PNBU00000000
P. rubra SCSIO 6842	+	**5.9**	10.57	GCF_001482385.1
P. rubra S2678	+	5.7	13.13	PNCH00000000
P. rubra ATCC 29570	+	6.0	12.47	GCF_000238295.2
P. luteoviolacea HI1	+	6.1	8.67	GCF_000814765.1
P. luteoviolacea H33	+	6.1	12.04	GCF_001625585.1
P. luteoviolacea IPB1	+	6.0	11.97	GCF_001696455.1
P. luteoviolacea S2607	+	6.0	14.54	GCF_001625575.1
P.luteoviolacea S4054	+	6.2	11.69	GCF_001750185.1
P. luteoviolacea S4060	+	5.9	11.43	GCF_001625705.1
P. luteoviolacea NCIMB 1944	+	6.3	12.83	GCF_001625565.1
P. luteoviolacea NCIMB 1942	+	5.4	8.97	GCF_001625595.1
P. luteoviolacea CPMOR-2	+	5.8	10	GCF_001625645.1
P. luteoviolacea CPMOR-1	+	6.0	9.48	GCF_001625685.1
P. luteoviolacea H33-S	+	6.1	11.84	GCF_001625695.1
P. ruthenica CP76	+	4.0	1.5	GCF_000336495.1
Pseudoalteromonas sp. SW0106-04	+	4.1	2.66	GCF_001293805.1
P. ruthenica S2756	+	4.0	2.73	SAMN08094581
P. ruthenica S2897	+	4.1	2.53	PNCG00000000
P. ruthenica S2899	+	4.1	2.52	PNCE00000000
P. ruthenica S4388	+	3.9	2.51	PNBS00000000
P. atlantica T6c	-	5.2	1.01	GCF_000014225.1
P. haloplanktis TAC125	-	3.9	1.41	GCF_000026085.1
P. distincta ANT/505	-	4.5	1.88	GCF_000212655.2
P. marina DSM 17587	-	4.2	0.26	GCF_000238335.2
P. haloplanktis ATCC 14393	-	5.0	1.3	GCF_000238355.1
Pseudoalteromonas arctica A 37-1-2	-	4.7	1.63	GCF_000238395.3
Pseudoalteromonas sp. BSi20429	-	4.5	1.35	GCF_000238895.1
Pseudoalteromonas sp. BSi20311	-	4.0	0.59	GCF_000239875.1
Pseudoalteromonas sp. BSi20495	-	4.8	4.41	GCF_000241185.1
Pseudoalteromonas sp. S8-8	-	4.9	1.33	GCF_000497875.1
Pseudoalteromonas sp. TAE56	-	4.6	1.13	GCF_000497955.1
Pseudoalteromonas sp. TB51	-	4.6	0.51	GCF_000498055.1
Pseudoalteromonas sp. TAB23	-	5.1	0.95	GCF_000498075.1
Pseudoalteromonas sp. TB64	-	4.8	1.82	GCF_000498095.1
Pseudoalteromonas sp. NW 4327	-	4.4	0.52	GCF_000508785.1
Pseudoalteromonas sp. 23 GOM-1509m	-	4.1	0.57	GCF_000518425.1
Pseudoalteromonas sp. SCSIO 11900	-	3.7	0.29	GCF_000576475.1
P. lipolytica SCSIO 04301	-	4.7	1.15	GCF_000576675.1
Pseudoalteromonas sp. A2	-	4.2	0.8	GCF_000738935.1
Pseudoalteromonas sp. ND6B	-	4.2	0.44	GCF_000764215.1
Pseudoalteromonas sp. PLSV	-	5.2	1	GCF_000767305.1
P. distincta ATCC 700518	-	4.5	0.48	GCF_000814675.1
P. tetradonis UCD-SED8	-	4.0	0.58	GCF_001298405.1

P. porphyrae UCD-SED9	-	4.8	2.9	GCF_001298415.1
P. lipolytica UCD-48B	-	4.6	1.47	GCF_001306915.1
Pseudoalteromonas sp. P1-8	-	4.5	1.49	GCF_001399985.1
Pseudoalteromonas sp. P1-11	-	4.4	1.53	GCF_001399995.1
Pseudoalteromonas sp. P1-25	-	4.4	0.42	GCF_001401805.1
P. translucida KMM 520	-	4.1	1.35	GCF_001465295.1
Pseudoalteromonas sp. H105	-	4.5	0.5	GCF_001469215.1
P. arabiensis JCM 17292	-	4.5	1.49	GCF_001550155.1
P. neustonica PAMC 28425	-	5.0	2.81	GCF_001653135.1
P. prydzensis DSM 14232	-	5.1	3.02	GCF_001661495.1
P. mariniglutinosa KCTC 22327	-	5.0	1.3	GCF_001662245.1
P. tetradonis CSB01KR	-	3.7	0.3	GCF_001723425.1
P. *haloplanktis* ATCC 700530	-	4.0	0.58	GCF_001924935.1
Pseudoalteromonas sp. SK20	-	4.1	0.15	GCF_001974845.1
Pseudoalteromonas sp. SK18	-	4.0	1.69	GCF_001974855.1
P. aliena EH1	-	4.6	3.99	GCF_001999225.1
Pseudoalteromonas sp. A601	-	4.9	2.2	GCF_002165575.1
P. nigrifaciens KMM 661	-	4.3	2.3	GCF_002221505.1
P. issachenkonii KMM 3549	-	4.1	0.56	GCF_002310795.1
P. tetradonis GFC	-	4.1	0.56	GCF_002310835.1
P. agarivorans DSM 14585	-	4.5	1.47	GCF_002310855.1
P. atlantica ECSMB14104	-	3.7	0.3	GCF_002374815.1
Pseudoalteromonas sp. 3D05	-	4.5	0.51	GCF_002723455.1
Pseudoalteromonas sp. T1lg75	-	3.6	1.5	GCF_002964705.1
Pseudoalteromonas sp. T1lg88	-	3.4	1.6	GCF_002964765.1
Pseudoalteromonas sp. T1lg48	-	3.6	1.52	GCF_002964785.1
P. carrageenovora IAM 12662	-	4.6	0.51	GCF_900239935.1
P. undina S1609	-	4.1	0.71	PNDJ00000000
P. undina S1612	-	4.0	0.46	PNDH00000000
P. lipolytica S1650	-	4.8	1.29	PNDF00000000
P. issachenkonii S1688	-	4.3	0.42	PNDE00000000
Pseudoalteromonas sp. S1727	-	5.0	1.22	PNDT00000000
P. issachenkonii S201	-	4.3	0.43	PNDR00000000
P. lipolytica S2721	-	4.5	1.37	PNCZ00000000
P. agarivorans S2893	-	4.3	0.43	PNCY00000000
P. issachenkonii S3173	-	4.2	0.44	PNCX00000000
Pseudoalteromonas sp. S3178	-	4.5	0.41	PNCW00000000
P. issachenkonii S3260	-	3.9	0.15	PNCV00000000
Pseudoalteromonas sp. S3431	-	4.2	3.3	GCF_000690035
Pseudoalteromonas sp. S3785	-	4.2	0.44	PNCT00000000
Pseudoalteromonas sp. S410	-	4.0	0.46	PNDW00000000
Pseudoalteromonas sp. S4389	-	4.8	0.23	PNCS00000000
P. shioyasakiensis S4491	-	4.5	1.36	PNCQ00000000
Pseudoalteromonas sp. S4492	-	4.6	1.33	PNCP00000000

P. issachenkonii S4741	-	3.8	0.16	PNCN00000000
P. marina S554	-	4.2	0.14	PNDV00000000
P. aliena S558	-	4.5	1.91	PNDU00000000
P. agarivorans S983	-	4.4	1.34	PNDL00000000
Pseudoalteromonas sp. SM9913	-	4.0	0.57	GCF_000184065.1
Pseudoalteromonas sp. BSi20652	-	4.2	0.45	GCF_000239855.1
Pseudoalteromonas sp. BSi20439	-	3.9	0.48	GCF_000241165.1
P. *agarivorans* S816	-	4.4	1.51	GCF_000363985.1
Pseudoalteromonas sp. TB13	-	4.7	0.98	GCF_000497915.1
Pseudoalteromonas sp. TB41	-	4.6	2.52	GCF_000497975.1
Pseudoalteromonas sp. TB25	-	4.6	0.4	GCF_000497995.1
Pseudoalteromonas sp. TAE79	-	5.0	0.6	GCF_000498015.1
Pseudoalteromonas sp. P1-30	-	4.3	1.54	GCF_001400005.1
Pseudoalteromonas sp. 13-15	-	4.1	0.26	GCF_001468205.2
Pseudoalteromonas sp. 10-33	-	4.2	0.55	GCF_001469195.1
Pseudoalteromonas sp. H103	-	4.4	0.53	GCF_001469205.1
Pseudoalteromonas sp. XI10	-	4.5	0.47	GCF_001469895.1
Pseudoalteromonas sp. MQS005	-	4.2	0.55	GCF_001661605.1
Pseudoalteromonas sp. EB27	-	4.5	1.44	GCF_001974875.1
P. *espejiana* DSM 9414	-	4.5	1.05	GCF_002221525.1
P. marina ECSMB14103	-	3.4	0.31	GCF_002407085.1
Pseudoalteromonas sp. 1_2015MBL_MicDiv	-	4.9	4.04	GCF_002407505.1
P. arctica MelAa3	-	4.5	1.46	GCF_002836095.1
Pseudoalteromonas sp. T1lg22	-	3.4	1.59	GCF_002964645.1
P. undina S1608	-	4.1	0.45	PNDK00000000
P. agarivorans S326	-	4.6	1.29	PNDQ00000000
P. undina S1610	-	3.9	0.46	PNDI00000000
P. undina DSM 6065	-	4.0	0.58	GCF_000238275.2
