# Supplementary material for: Marine Chitinolytic Pseudoalteromonas Represents an Untapped Reservoir of Bioactive Potential
Source: mSystems. 2019 Jun 18;4(4):e00060-19. doi: 10.1128/mSystems.00060-19 (PMC6581688; doi:10.1128/mSystems.00060-19)
Supplement: TABLE S2 [file mSystems.00060-19-st002.pdf]

Table S2

Strain	Pigment	Chitin degradation	Inhibition of *V. anguillarum* on different carbon sources			
			Glucose	*N*-acetylglucosamine	Colloidal chitin	Crystalline chitin
S201	-	+	-	-	-	-
S326	-	-	-	-	-	-
S410	-	-	-	-	-	-
S554	-	-	-	-	-	-
S558	-	+	+	+	+	+
S816	-	-	-	-	-	-
S983	-	-	-	-	-	-
S1093	+	+	+	+	+	+
S1189	+	+	+	+	+	+
S1608	-	+	-	-	-	-
S1609	-	+	-	-	-	-
S1610	-	+	-	-	-	-
S1612	-	+	-	-	-	-
S1650	-	+	-	-	-	-
S1688	-	+	-	-	-	-
S1727	-	+	-	-	-	-
S1946	+	+	+	+	+	+
S2049	+	+	+	+	+	+
S2231	+	+	+	+	+	+
S2471	+	+	+	+	+	+
S2599	+	+	+	+	+	+
S2607	+	+	+	+	+	+
S2676	+	+	+	+	+	+
S2678	+	+	+	+	+	+
S2721	-	+	-	-	-	-
S2724	+	+	+	+	+	+
S2755	+	+	+	+	+	+
S2756	+	+	+	+	+	+
S2893	-	-	+	+	+	+
S2897	+	+	+	+	+	+
S2899	+	+	+	+	+	+
S3173	-	+	-	-	-	-
S3178	-	-	-	-	-	-
S3260	-	-	-	-	-	-
S3431	-	-	-	-	-	-
S3655	+	+	+	+	+	+
S3663	+	+	+	+	+	+
S3785	-	+	-	-	-	-
S3895	+	+	+	+	+	+
S3898	+	+	+	+	+	+
S4048	+	+	+	+	+	+
S4054	+	+	+	+	+	+

Table S2

S4059	+	+	+	+	+	+
S4060	+	+	+	+	+	+
S4388	+	+	+	+	+	+
S4389	-	+	-	-	-	-
S4491	-	+	-	-	-	-
S4492	-	+	-	-	-	-
S4498	-	+	+	+	+	+
S4741	-	-	-	-	-	-
