# Supplementary material for: Marine Chitinolytic Pseudoalteromonas Represents an Untapped Reservoir of Bioactive Potential
Source: mSystems. 2019 Jun 18;4(4):e00060-19. doi: 10.1128/mSystems.00060-19 (PMC6581688; doi:10.1128/mSystems.00060-19)
Supplement: TABLE S3 [file mSystems.00060-19-st003.pdf]

Table S3

	**MDS1**	**MDS2**
GH1	-0.42117	-0.26314
GH2	-0.38043	-0.24253
GH3	-0.22242	-0.43467
GH4	-0.13611	0.175117
GH5	0.138036	-0.18002
GH8	-0.31839	-0.10265
GH9	-0.01321	0.001193
GH10	-0.01751	-0.00147
GH11	-0.00058	-0.00067
**GH13**	**-0.72773**	**-0.88561**
GH15	0.127772	-0.11234
**GH16**	**-0.25866**	**-0.78953**
**GH18**	**2.463076**	**-0.36018**
**GH19**	**0.541572**	**-0.1021**
**GH20**	**1.066747**	**-0.4403**
**GH23**	**0.611899**	**-0.00547**
**GH24**	**0.976886**	**-0.19688**
GH25	0.181158	0.015096
GH26	-0.0069	0.002221
GH28	-0.05528	-0.13709
GH29	-0.02057	-0.15065
GH31	-0.16693	-0.03702
GH32	-0.11153	-0.19814
GH33	0.004853	0.046578
GH36	-0.32145	0.002166
GH37	0.112189	-0.13264
GH39	-0.01437	-0.10174
GH42	-0.07723	-0.09341
GH43	-0.41987	-1.74226
**GH50**	**-0.27421**	**-0.53986**
GH63	-0.09032	-0.11359
GH68	-0.02308	0.00294
GH73	0.030718	-0.04107
**GH74**	**1.815976**	**0.09098**
GH76	-0.00494	-0.09532
GH77	0.027596	-0.04329
**GH78**	**-0.07722**	**-0.56227**
GH82	-0.04677	-0.00788
GH86	-0.09753	-0.12533
GH87	-0.00354	-0.01373
GH88	-0.0157	-0.12488
**GH92**	**0.548718**	**-0.11532**

GH93	0.008992	0.001335
GH94	-0.03951	-0.04344
GH97	-0.04639	-0.18816
GH99	0.008146	0.004824
GH102	-0.00202	0.015596
GH103	0.007846	-0.0945
GH105	-0.07569	-0.30576
GH106	-0.02447	-0.21043
GH107	-0.03292	-0.0316
GH108	0.075344	-0.04846
**GH109**	**-0.00086**	**-0.927**
GH110	-0.01751	-0.00147
GH113	-0.00777	0.004529
GH114	0.009683	0.11058
GH117	-0.23679	-0.39671
GH127	-0.0534	-0.25332
GH130	-0.0069	0.002221
GH135	-0.02072	-0.07244
GH136	0.187281	-0.01119
GH141	0.009374	-0.02561
GH145	0.034633	-0.12678
