# Supplementary material for: Marine Chitinolytic Pseudoalteromonas Represents an Untapped Reservoir of Bioactive Potential
Source: mSystems. 2019 Jun 18;4(4):e00060-19. doi: 10.1128/mSystems.00060-19 (PMC6581688; doi:10.1128/mSystems.00060-19)
Supplement: FIG S2 [file mSystems.00060-19-sf002.pdf]

Figure S2

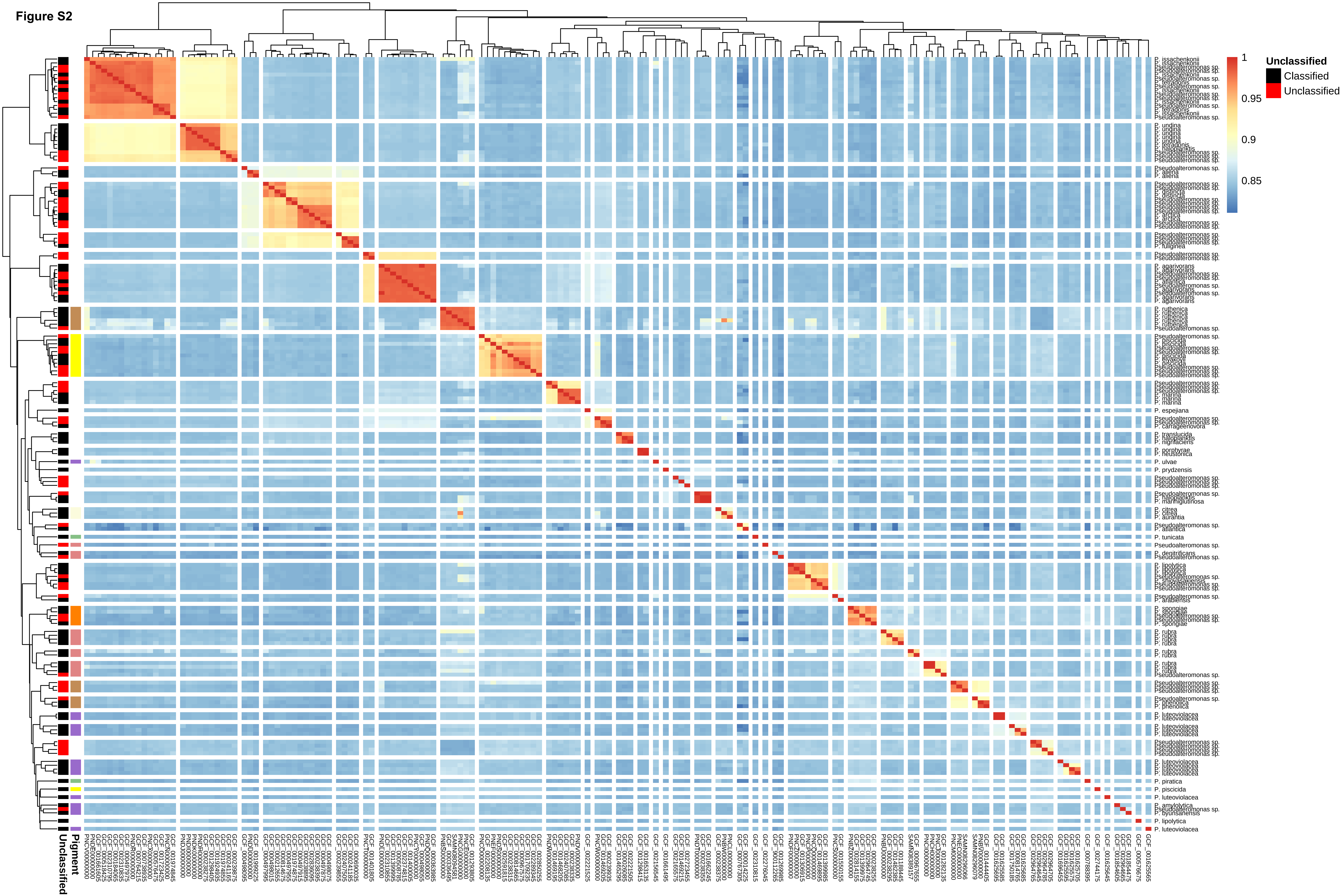

Unclassified
Classified
Unclassified
1
0.95
0.9
0.85
Pigment
Unclassified
P. espejiana
P. ulvae
P. prydzensis
P. tunicata
Pseudoalteromonas sp.
P. piratica
P. piscicida
P. luteoviolacea
P. lipolytica
P. luteoviolacea
